# Hand, foot, and mouth syndrome in an immunocompetent adult: a case report

**DOI:** 10.1186/1756-0500-6-441

**Published:** 2013-11-03

**Authors:** Bárbara Flor de Lima, João Silva, Ana Catarina Rodrigues, Ana Grilo, Nuno Riso, Manuel Vaz Riscado

**Affiliations:** 1 Departments of Infectious Diseases, Hospital de Curry Cabral, Rua da Beneficência n.º 8 1069-166, Lisbon, Portugal; 2 Departments of Auto-immunity - Internal Medicine, Hospital de Curry Cabral, Lisbon, Portugal

**Keywords:** Hand, foot, and mouth syndrome, Myopericarditis, Coxsackie A9, Celiac disease

## Abstract

**Background:**

Hand, foot, and mouth syndrome (HFMS) is a common acute illness. It is characterized by mild clinical symptoms including fever, blisters, and sores in the mouth and on the palms and soles following a 3- to 7-day incubation period. This syndrome is rarely seen in adults.

**Case presentation:**

A 35-year-old male Caucasian patient had a history of multiple episodes of acute pharyngitis, hypertension, hypercholesterolemia, and occasional abdominal pain. He presented with polyarthralgia in the knees and hands and odynophagia, followed by fever, oral mucosal aphthous lesions, and vesicles on the palms and soles. Three weeks after presentation, he was admitted to the emergency room with acute myocarditis. The in-hospital evaluation revealed positive serology for coxsackie A9 (1:160), positive anti-transglutaminase and anti-gliadin antibodies, normal immunoglobulins, and human immunodeficiency virus negativity.

**Conclusion:**

We herein describe a case of HFMS that was associated with coxsackie A9 infection complicated by acute myocarditis. Although an association between celiac disease and HFMS has not been described, this patient’s immunologic disruption could have favored the development of infection and ultimately HFMS.

## Background

Hand, foot, and mouth syndrome (HFMS) mainly affects children [1,2]. It is characterized by mild clinical symptoms including fever and blisters and sores in the mouth and on the palms and soles following a 3- to 7-day incubation period, with recovery in 7 to 10 days [3]. It is caused by infection with an enterovirus, mainly enterovirus 71 (EV71) and coxsackie A16 (CA16) [4]. In a minority of cases, isolation of the agent is not possible [1]. We herein report an atypical presentation of HFMS in an immunocompetent adult.

## Case presentation

A 35-year-old Caucasian male patient who worked as an environmental engineer had a medical history of recurrent episodes of acute pharyngitis, essential hypertension, hypercholesterolemia, and occasional abdominal pain and bloating. He had no recent travel history. His family history included immunoglobulin (Ig) A deficiency in his 18-month-old son and a several-year history of nonspecific colitis in his father. Regular medications were nifedipine CR (30 mg/day) and rosuvastatin (5 mg/day).

**Figure 1:**
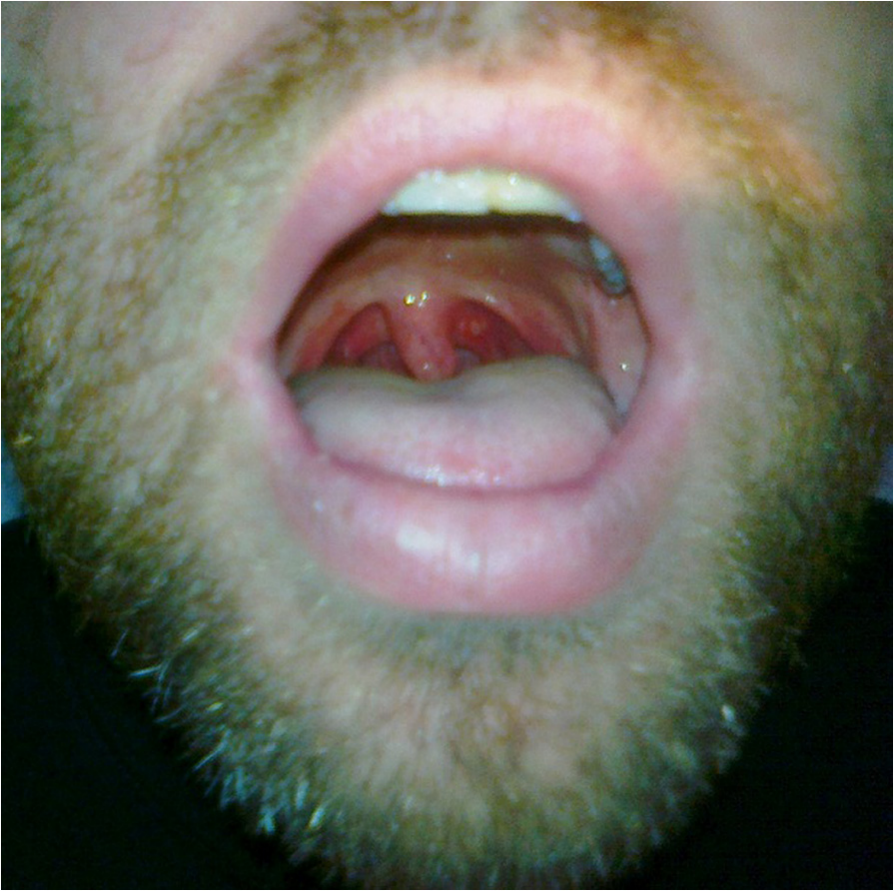
HFMS: oral vesicles.

**Figure 2:**
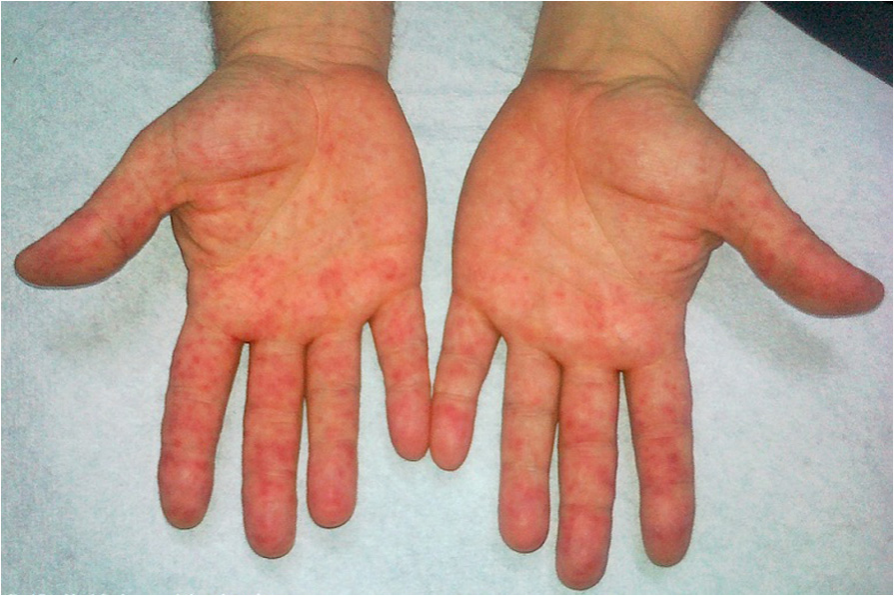
HFMS: vesicular rash on palms.

The patient initially presented to his family physician with a symmetrical polyarthralgia involving his knees and hands, odynophagia, temporal headache, retro-ocular pain, and an intermittent fever (maximum of 38°C). He was initially managed with an antibiotic (azithromycin) and anti-inflammatory agents without resolution of symptoms. One week later, he developed oral mucosal lesions and a vesicular rash on his palms and soles (Figures 1 and 2). In April 2011, 3 weeks after the initial presentation, he was admitted to the emergency room at the Curry Cabral Hospital (Lisbon) with compressive retrosternal pain, without dyspnea or a productive cough. The odynophagia was persistent, with worsening of the headache and arthralgia.

The emergency staff noted nonexudative oropharyngitis and tender cervical lymphadenopathy with normal cardiorespiratory examination findings and absence of a pericardial friction rub. He had abdominal tenderness in the right lower quadrant without guarding. There were no vesicles on the palms of his hands or soles of his feet, but the skin had a scalded appearance. There was slight ST-segment depression on the inferior leads (II, III, and aVF) of the electrocardiogram (ECG) and mildly increased levels of troponin I and creatine kinase-MB fraction. The diagnosis of myopericarditis was considered. He started ibuprofen at 400 mg three times a day and was admitted for further workup.

**Figure 3:**
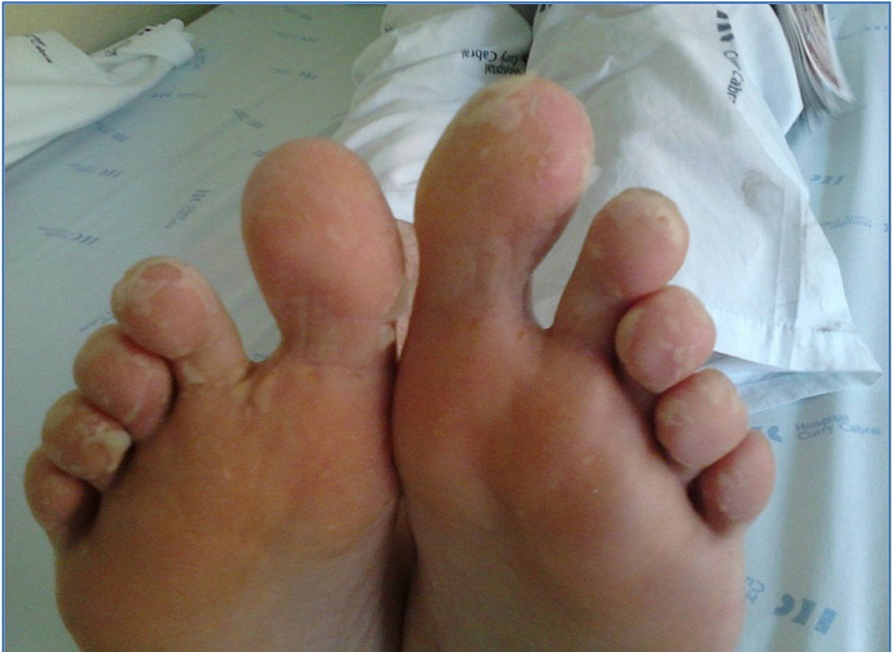
HFMS: skin with a scaly appearance, 1 week after the vesicular rash.

As an inpatient, his condition slowly improved. His fever resolved and he was discharged with only slight fatigue during strenuous exercise. The skin vesicles disappeared and progressed to scaliness (Figure 3).

Laboratory examination showed positive serology for coxsackie A9 (IgM with a titer of 1:160) 1 week after the disappearance of the vesicular rash. It is assumed that a titer of >1:80 is predictive of a diagnosis of the infection in question. In addition, he was positive for IgA anti-transglutaminase 25 (<20 U/mL) and IgA anti-gliadin 77 (<25 U/mL) antibodies, with higher titers during follow-up after discharge (59 and 60 U/mL, respectively). He had normal immunoglobulin levels and was negative for antinuclear antibody (ANA), anti-DNA antibody (dsDNA), and p- and c-anti-neutrophil cytoplasmic antibody (ANCA). Serology for viral hepatitis and human immunodeficiency virus were negative. Endoscopy revealed a normal appearance of the gastric and duodenal mucosae, and biopsy showed slight mucosal architecture disruption.

An echocardiogram showed a normal left ventricle, slightly hypertrophic interventricular septum, normal systolic function (ejection fraction of 60%), and left atrium in the upper limit of the normal range. The ECG findings and cardiac biomarker levels returned to normal after 5 days.

To ameliorate the abdominal pain and bloating, a gluten-free diet was introduced, and this proved to be helpful with normalization of the anti-transglutaminase and anti-gliadin antibodies 6 months later.

## Conclusions

To the best of our knowledge, HFMS in adults has rarely been reported [1,5-7]. Previously reported cases had a typical presentation with mucocutaneous involvement, and one case was complicated by arthritis. Even in reports of HFMS epidemics in children, only one case of a generalized neonatal coxsackie A9 infection presenting with massive pleural effusion has been reported [1,2,8].

We herein reported a case of HFMS complicated by myopericarditis, which has not been previously described [1]. Coxsackie A9 is not the most common cause of HFMS among the epidemics that have occurred worldwide. Outbreaks due to EV71 were seen in the Asia Pacific region in 1997 and in Taiwan in 1998 [4]. In 2007, the same was observed in China, with seasonal outbreaks in later years [9].

The coxsackie B group is more commonly described as a cause of myopericarditis, although coxsackie A9 is also cardiotropic. A neurologic presentation and hepatic involvement have been reported in association with the latter virus [10-12].

Many researchers are now studying risk factors for HFMS. Infectious disease control exercises and good hygiene practices are important to prevent the transmission of HFMS. In the present case, there was no close contact with individuals with similar symptoms. Geographic factors have been associated with incident cases as well as mortality, with severe cases associated with rural areas and more common in patients under 6 years of age and with a low level of medical care [1]. HFMS is reportedly more common in rainy seasons according to geographical location [5]. While outbreaks usually occur in summer or early fall in temperate countries, enterovirus surveillance in the US showed that EV71 and CA16 have an endemic pattern and that the majority of cases are reported during warmer seasons, between June and October [13]. In spite of these seasonal outbreaks, our case was reported in April and was not related to rainy weather or high temperatures. It was not possible to perform molecular diagnosis (RT-PCR) or viral culture in our case because the patient was admitted after the development of viremia and a vesicular rash.

There are no studies showing a relationship between celiac disease and HFMS; however, bearing its immunologic disruption [14], it may be associated with a higher risk for infection or a more symptomatic presentation than commonly seen in adults.

## Consent

Written informed consent was obtained from the patient for publication of this case report and any accompanying images. A copy of the written consent is available for review by the Editor-in-Chief of this journal.

### Ethical approval

This case report was approved by the Hospital Ethical Commission.

## Competing interests

The authors declare that they have no competing interests.

## Authors’ contributions

BFL, JS, and ACR were responsible for the writing of the manuscript. AG was responsible the case management and work up and manuscript revision. NR and MVR were responsible for the manuscript revision. All authors read and approved the final manuscript.
